# Measurements of the branching fractions of $B^{+} \to p \bar{p} K^{+}$ decays

**DOI:** 10.1140/epjc/s10052-013-2462-2

**Published:** 2013-06-13

**Authors:** The LHCb Collaboration, R. Aaij, C. Abellan Beteta, A. Adametz, B. Adeva, M. Adinolfi, C. Adrover, A. Affolder, Z. Ajaltouni, J. Albrecht, F. Alessio, M. Alexander, S. Ali, G. Alkhazov, P. Alvarez Cartelle, A. A. Alves, S. Amato, Y. Amhis, L. Anderlini, J. Anderson, R. Andreassen, R. B. Appleby, O. Aquines Gutierrez, F. Archilli, A. Artamonov, M. Artuso, E. Aslanides, G. Auriemma, S. Bachmann, J. J. Back, C. Baesso, V. Balagura, W. Baldini, R. J. Barlow, C. Barschel, S. Barsuk, W. Barter, Th. Bauer, A. Bay, J. Beddow, I. Bediaga, S. Belogurov, K. Belous, I. Belyaev, E. Ben-Haim, M. Benayoun, G. Bencivenni, S. Benson, J. Benton, A. Berezhnoy, R. Bernet, M.-O. Bettler, M. van Beuzekom, A. Bien, S. Bifani, T. Bird, A. Bizzeti, P. M. Bjørnstad, T. Blake, F. Blanc, C. Blanks, J. Blouw, S. Blusk, A. Bobrov, V. Bocci, A. Bondar, N. Bondar, W. Bonivento, S. Borghi, A. Borgia, T. J. V. Bowcock, E. Bowen, C. Bozzi, T. Brambach, J. van den Brand, J. Bressieux, D. Brett, M. Britsch, T. Britton, N. H. Brook, H. Brown, I. Burducea, A. Bursche, J. Buytaert, S. Cadeddu, O. Callot, M. Calvi, M. Calvo Gomez, A. Camboni, P. Campana, A. Carbone, G. Carboni, R. Cardinale, A. Cardini, H. Carranza-Mejia, L. Carson, K. Carvalho Akiba, G. Casse, M. Cattaneo, Ch. Cauet, M. Charles, Ph. Charpentier, P. Chen, N. Chiapolini, M. Chrzaszcz, K. Ciba, X. Cid Vidal, G. Ciezarek, P. E. L. Clarke, M. Clemencic, H. V. Cliff, J. Closier, C. Coca, V. Coco, J. Cogan, E. Cogneras, P. Collins, A. Comerma-Montells, A. Contu, A. Cook, M. Coombes, S. Coquereau, G. Corti, B. Couturier, G. A. Cowan, D. Craik, S. Cunliffe, R. Currie, C. D’Ambrosio, P. David, P. N. Y. David, I. De Bonis, K. De Bruyn, S. De Capua, M. De Cian, J. M. De Miranda, L. De Paula, W. De Silva, P. De Simone, D. Decamp, M. Deckenhoff, H. Degaudenzi, L. Del Buono, C. Deplano, D. Derkach, O. Deschamps, F. Dettori, A. Di Canto, J. Dickens, H. Dijkstra, M. Dogaru, F. Domingo Bonal, S. Donleavy, F. Dordei, A. Dosil Suárez, D. Dossett, A. Dovbnya, F. Dupertuis, R. Dzhelyadin, A. Dziurda, A. Dzyuba, S. Easo, U. Egede, V. Egorychev, S. Eidelman, D. van Eijk, S. Eisenhardt, U. Eitschberger, R. Ekelhof, L. Eklund, I. El Rifai, Ch. Elsasser, D. Elsby, A. Falabella, C. Färber, G. Fardell, C. Farinelli, S. Farry, V. Fave, D. Ferguson, V. Fernandez Albor, F. Ferreira Rodrigues, M. Ferro-Luzzi, S. Filippov, C. Fitzpatrick, M. Fontana, F. Fontanelli, R. Forty, O. Francisco, M. Frank, C. Frei, M. Frosini, S. Furcas, E. Furfaro, A. Gallas Torreira, D. Galli, M. Gandelman, P. Gandini, Y. Gao, J. Garofoli, P. Garosi, J. Garra Tico, L. Garrido, C. Gaspar, R. Gauld, E. Gersabeck, M. Gersabeck, T. Gershon, Ph. Ghez, V. Gibson, V. V. Gligorov, C. Göbel, D. Golubkov, A. Golutvin, A. Gomes, H. Gordon, M. Grabalosa Gándara, R. Graciani Diaz, L. A. Granado Cardoso, E. Graugés, G. Graziani, A. Grecu, E. Greening, S. Gregson, O. Grünberg, B. Gui, E. Gushchin, Yu. Guz, T. Gys, C. Hadjivasiliou, G. Haefeli, C. Haen, S. C. Haines, S. Hall, T. Hampson, S. Hansmann-Menzemer, N. Harnew, S. T. Harnew, J. Harrison, P. F. Harrison, T. Hartmann, J. He, V. Heijne, K. Hennessy, P. Henrard, J. A. Hernando Morata, E. van Herwijnen, E. Hicks, D. Hill, M. Hoballah, C. Hombach, P. Hopchev, W. Hulsbergen, P. Hunt, T. Huse, N. Hussain, D. Hutchcroft, D. Hynds, V. Iakovenko, P. Ilten, R. Jacobsson, A. Jaeger, E. Jans, F. Jansen, P. Jaton, F. Jing, M. John, D. Johnson, C. R. Jones, B. Jost, M. Kaballo, S. Kandybei, M. Karacson, T. M. Karbach, I. R. Kenyon, U. Kerzel, T. Ketel, A. Keune, B. Khanji, O. Kochebina, I. Komarov, R. F. Koopman, P. Koppenburg, M. Korolev, A. Kozlinskiy, L. Kravchuk, K. Kreplin, M. Kreps, G. Krocker, P. Krokovny, F. Kruse, M. Kucharczyk, V. Kudryavtsev, T. Kvaratskheliya, V. N. La Thi, D. Lacarrere, G. Lafferty, A. Lai, D. Lambert, R. W. Lambert, E. Lanciotti, G. Lanfranchi, C. Langenbruch, T. Latham, C. Lazzeroni, R. Le Gac, J. van Leerdam, J.-P. Lees, R. Lefèvre, A. Leflat, J. Lefrançois, O. Leroy, Y. Li, L. Li Gioi, M. Liles, R. Lindner, C. Linn, B. Liu, G. Liu, J. von Loeben, J. H. Lopes, E. Lopez Asamar, N. Lopez-March, H. Lu, J. Luisier, H. Luo, F. Machefert, I. V. Machikhiliyan, F. Maciuc, O. Maev, S. Malde, G. Manca, G. Mancinelli, N. Mangiafave, U. Marconi, R. Märki, J. Marks, G. Martellotti, A. Martens, L. Martin, A. Martín Sánchez, M. Martinelli, D. Martinez Santos, D. Martins Tostes, A. Massafferri, R. Matev, Z. Mathe, C. Matteuzzi, M. Matveev, E. Maurice, A. Mazurov, J. McCarthy, R. McNulty, B. Meadows, F. Meier, M. Meissner, M. Merk, D. A. Milanes, M.-N. Minard, J. Molina Rodriguez, S. Monteil, D. Moran, P. Morawski, R. Mountain, I. Mous, F. Muheim, K. Müller, R. Muresan, B. Muryn, B. Muster, P. Naik, T. Nakada, R. Nandakumar, I. Nasteva, M. Needham, N. Neufeld, A. D. Nguyen, T. D. Nguyen, C. Nguyen-Mau, M. Nicol, V. Niess, R. Niet, N. Nikitin, T. Nikodem, S. Nisar, A. Nomerotski, A. Novoselov, A. Oblakowska-Mucha, V. Obraztsov, S. Oggero, S. Ogilvy, O. Okhrimenko, R. Oldeman, M. Orlandea, J. M. Otalora Goicochea, P. Owen, B. K. Pal, A. Palano, M. Palutan, J. Panman, A. Papanestis, M. Pappagallo, C. Parkes, C. J. Parkinson, G. Passaleva, G. D. Patel, M. Patel, G. N. Patrick, C. Patrignani, C. Pavel-Nicorescu, A. Pazos Alvarez, A. Pellegrino, G. Penso, M. Pepe Altarelli, S. Perazzini, D. L. Perego, E. Perez Trigo, A. Pérez-Calero Yzquierdo, P. Perret, M. Perrin-Terrin, G. Pessina, K. Petridis, A. Petrolini, A. Phan, E. Picatoste Olloqui, B. Pietrzyk, T. Pilař, D. Pinci, S. Playfer, M. Plo Casasus, F. Polci, G. Polok, A. Poluektov, E. Polycarpo, D. Popov, B. Popovici, C. Potterat, A. Powell, J. Prisciandaro, V. Pugatch, A. Puig Navarro, W. Qian, J. H. Rademacker, B. Rakotomiaramanana, M. S. Rangel, I. Raniuk, N. Rauschmayr, G. Raven, S. Redford, M. M. Reid, A. C. dos Reis, S. Ricciardi, A. Richards, K. Rinnert, V. Rives Molina, D. A. Roa Romero, P. Robbe, E. Rodrigues, P. Rodriguez Perez, G. J. Rogers, S. Roiser, V. Romanovsky, A. Romero Vidal, J. Rouvinet, T. Ruf, H. Ruiz, G. Sabatino, J. J. Saborido Silva, N. Sagidova, P. Sail, B. Saitta, C. Salzmann, B. Sanmartin Sedes, M. Sannino, R. Santacesaria, C. Santamarina Rios, E. Santovetti, M. Sapunov, A. Sarti, C. Satriano, A. Satta, M. Savrie, D. Savrina, P. Schaack, M. Schiller, H. Schindler, S. Schleich, M. Schlupp, M. Schmelling, B. Schmidt, O. Schneider, A. Schopper, M.-H. Schune, R. Schwemmer, B. Sciascia, A. Sciubba, M. Seco, A. Semennikov, K. Senderowska, I. Sepp, N. Serra, J. Serrano, P. Seyfert, M. Shapkin, I. Shapoval, P. Shatalov, Y. Shcheglov, T. Shears, L. Shekhtman, O. Shevchenko, V. Shevchenko, A. Shires, R. Silva Coutinho, T. Skwarnicki, N. A. Smith, E. Smith, M. Smith, K. Sobczak, M. D. Sokoloff, F. J. P. Soler, F. Soomro, D. Souza, B. Souza De Paula, B. Spaan, A. Sparkes, P. Spradlin, F. Stagni, S. Stahl, O. Steinkamp, S. Stoica, S. Stone, B. Storaci, M. Straticiuc, U. Straumann, V. K. Subbiah, S. Swientek, V. Syropoulos, M. Szczekowski, P. Szczypka, T. Szumlak, S. T’Jampens, M. Teklishyn, E. Teodorescu, F. Teubert, C. Thomas, E. Thomas, J. van Tilburg, V. Tisserand, M. Tobin, S. Tolk, D. Tonelli, S. Topp-Joergensen, N. Torr, E. Tournefier, S. Tourneur, M. T. Tran, M. Tresch, A. Tsaregorodtsev, P. Tsopelas, N. Tuning, M. Ubeda Garcia, A. Ukleja, D. Urner, U. Uwer, V. Vagnoni, G. Valenti, R. Vazquez Gomez, P. Vazquez Regueiro, S. Vecchi, J. J. Velthuis, M. Veltri, G. Veneziano, M. Vesterinen, B. Viaud, D. Vieira, X. Vilasis-Cardona, A. Vollhardt, D. Volyanskyy, D. Voong, A. Vorobyev, V. Vorobyev, C. Voß, H. Voss, R. Waldi, R. Wallace, S. Wandernoth, J. Wang, D. R. Ward, N. K. Watson, A. D. Webber, D. Websdale, M. Whitehead, J. Wicht, J. Wiechczynski, D. Wiedner, L. Wiggers, G. Wilkinson, M. P. Williams, M. Williams, F. F. Wilson, J. Wishahi, M. Witek, S. A. Wotton, S. Wright, S. Wu, K. Wyllie, Y. Xie, F. Xing, Z. Xing, Z. Yang, R. Young, X. Yuan, O. Yushchenko, M. Zangoli, M. Zavertyaev, F. Zhang, L. Zhang, W. C. Zhang, Y. Zhang, A. Zhelezov, L. Zhong, A. Zvyagin

**Affiliations:** 1 CERN, 1211 Geneva 23, Switzerland; 2 Centro Brasileiro de Pesquisas Físicas (CBPF), Rio de Janeiro, Brazil; 3 Universidade Federal do Rio de Janeiro (UFRJ), Rio de Janeiro, Brazil; 4 Center for High Energy Physics, Tsinghua University, Beijing, China; 5 LAPP, Université de Savoie, CNRS/IN2P3, Annecy-Le-Vieux, France; 6 Clermont Université, Université Blaise Pascal, CNRS/IN2P3, LPC, Clermont-Ferrand, France; 7 CPPM, Aix-Marseille Université, CNRS/IN2P3, Marseille, France; 8 LAL, Université Paris-Sud, CNRS/IN2P3, Orsay, France; 9 LPNHE, Université Pierre et Marie Curie, Université Paris Diderot, CNRS/IN2P3, Paris, France; 10 Fakultät Physik, Technische Universität Dortmund, Dortmund, Germany; 11 Max-Planck-Institut für Kernphysik (MPIK), Heidelberg, Germany; 12 Physikalisches Institut, Ruprecht-Karls-Universität Heidelberg, Heidelberg, Germany; 13 School of Physics, University College Dublin, Dublin, Ireland; 14 Sezione INFN di Bari, Bari, Italy; 15 Sezione INFN di Bologna, Bologna, Italy; 16 Sezione INFN di Cagliari, Cagliari, Italy; 17 Sezione INFN di Ferrara, Ferrara, Italy; 18 Sezione INFN di Firenze, Firenze, Italy; 19 Laboratori Nazionali dell’INFN di Frascati, Frascati, Italy; 20 Sezione INFN di Genova, Genova, Italy; 21 Sezione INFN di Milano Bicocca, Milano, Italy; 22 Sezione INFN di Roma Tor Vergata, Roma, Italy; 23 Sezione INFN di Roma La Sapienza, Roma, Italy; 24 Henryk Niewodniczanski Institute of Nuclear Physics, Polish Academy of Sciences, Kraków, Poland; 25 AGH University of Science and Technology, Kraków, Poland; 26 National Center for Nuclear Research (NCBJ), Warsaw, Poland; 27 Horia Hulubei National Institute of Physics and Nuclear Engineering, Bucharest-Magurele, Romania; 28 Petersburg Nuclear Physics Institute (PNPI), Gatchina, Russia; 29 Institute of Theoretical and Experimental Physics (ITEP), Moscow, Russia; 30 Institute of Nuclear Physics, Moscow State University (SINP MSU), Moscow, Russia; 31 Institute for Nuclear Research of the Russian Academy of Sciences (INR RAN), Moscow, Russia; 32 Budker Institute of Nuclear Physics (SB RAS) and Novosibirsk State University, Novosibirsk, Russia; 33 Institute for High Energy Physics (IHEP), Protvino, Russia; 34 Universitat de Barcelona, Barcelona, Spain; 35 Universidad de Santiago de Compostela, Santiago de Compostela, Spain; 36 European Organization for Nuclear Research (CERN), Geneva, Switzerland; 37 Ecole Polytechnique Fédérale de Lausanne (EPFL), Lausanne, Switzerland; 38 Physik-Institut, Universität Zürich, Zürich, Switzerland; 39 Nikhef National Institute for Subatomic Physics, Amsterdam, The Netherlands; 40 Nikhef National Institute for Subatomic Physics and VU University Amsterdam, Amsterdam, The Netherlands; 41 NSC Kharkiv Institute of Physics and Technology (NSC KIPT), Kharkiv, Ukraine; 42 Institute for Nuclear Research of the National Academy of Sciences (KINR), Kyiv, Ukraine; 43 University of Birmingham, Birmingham, United Kingdom; 44 H.H. Wills Physics Laboratory, University of Bristol, Bristol, United Kingdom; 45 Cavendish Laboratory, University of Cambridge, Cambridge, United Kingdom; 46 Department of Physics, University of Warwick, Coventry, United Kingdom; 47 STFC Rutherford Appleton Laboratory, Didcot, United Kingdom; 48 School of Physics and Astronomy, University of Edinburgh, Edinburgh, United Kingdom; 49 School of Physics and Astronomy, University of Glasgow, Glasgow, United Kingdom; 50 Oliver Lodge Laboratory, University of Liverpool, Liverpool, United Kingdom; 51 Imperial College London, London, United Kingdom; 52 School of Physics and Astronomy, University of Manchester, Manchester, United Kingdom; 53 Department of Physics, University of Oxford, Oxford, United Kingdom; 54 Syracuse University, Syracuse, NY United States; 55 Pontifícia Universidade Católica do Rio de Janeiro (PUC-Rio), Rio de Janeiro, Brazil; 56 Institut für Physik, Universität Rostock, Rostock, Germany; 57 Institute of Information Technology, COMSATS, Lahore, Pakistan; 58 University of Cincinnati, Cincinnati, OH United States

## Abstract

The branching fractions of the decay $B^{+} \to p \bar{p} K^{+}$ for different intermediate states are measured using data, corresponding to an integrated luminosity of 1.0 fb^−1^, collected by the LHCb experiment. The total branching fraction, its charmless component $(M_{p\bar{p}}<2.85~\text {GeV}/c^{2})$ and the branching fractions via the resonant $c\bar{c}$ states *η*_*c*_(1*S*) and *ψ*(2*S*) relative to the decay via a *J*/*ψ* intermediate state are  Upper limits on the *B* ^+^ branching fractions into the *η*_*c*_(2*S*) meson and into the charmonium-like states *X*(3872) and *X*(3915) are also obtained.

## Introduction

The $B^{+} \to p \bar{p} K^{+}$ decay^1^ offers a clean environment to study $c\bar{c}$ states and charmonium-like mesons that decay to $p\bar{p}$ and excited $\bar{ \varLambda }$ baryons that decay to $\bar{p} K^{+}$, and to search for glueballs or exotic states. The presence of $p \bar{p}$ in the final state allows intermediate states of any quantum numbers to be studied and the existence of the charged kaon in the final state significantly enhances the signal to background ratio in the selection procedure. Measurements of intermediate charmonium-like states, such as the *X*(3872), are important to clarify their nature [1, 2] and to determine their partial width to $p\bar{p}$, which is crucial to predict the production rate of these states in dedicated experiments [3]. BaBar and Belle have previously measured the $B^{+} \to p \bar{p} K^{+}$ branching fraction, including contributions from the *J*/*ψ* and *η*_*c*_(1*S*) intermediate states [4, 5]. The data sample, corresponding to an integrated luminosity of 1.0 fb^−1^, collected by LHCb at $\sqrt{s}=7~\text {TeV}$ allows the study of substructures in the $B^{+}\to p\bar{p} K^{+}$ decays with a sample ten times larger than those available at previous experiments.

In this paper we report measurements of the ratios of branching fractions

1 $$ \mathcal{R}({\rm mode}) =\frac{\mathcal{B}(B^{+} \to{\rm mode}\to p\bar{p} K^{+})}{\mathcal{B}(B^{+}\to J/\psi K^{+}\to p\bar{p} K^{+})}, $$

where “mode” corresponds to the intermediate *η*_*c*_(1*S*), *ψ*(2*S*), *η*_*c*_(2*S*), *χ*_*c*0_(1*P*), *h*_*c*_(1*P*), *X*(3872) or *X*(3915) states, together with a kaon.

## Detector and software

The LHCb detector [6] is a single-arm forward spectrometer covering the pseudorapidity range 2<*η*<5, designed for the study of particles containing *b* or *c* quarks. The detector includes a high precision tracking system consisting of a silicon-strip vertex detector surrounding the *pp* interaction region, a large-area silicon-strip detector located upstream of a dipole magnet with a bending power of about $4{\rm\,Tm}$, and three stations of silicon-strip detectors and straw drift-tubes placed downstream. The combined tracking system has momentum (*p*) resolution Δ*p*/*p* that varies from 0.4 % at 5 GeV/*c* to 0.6 % at 100 GeV/*c*, and impact parameter resolution of 20 μm for tracks with high transverse momentum ($p_{\rm T}$). Charged hadrons are identified using two ring-imaging Cherenkov (RICH) detectors. Photon, electron and hadron candidates are identified by a calorimeter system consisting of scintillating-pad and pre-shower detectors, an electromagnetic calorimeter and a hadronic calorimeter. Muons are identified by a system composed of alternating layers of iron and multiwire proportional chambers.

The trigger [7] consists of a hardware stage, based on information from the calorimeter and muon systems, followed by a software stage where candidates are fully reconstructed. The hardware trigger selects hadrons with high transverse energy in the calorimeter. The software trigger requires a two-, three- or four-track secondary vertex with a high $p_{\rm T}$ sum of the tracks and a significant displacement from the primary *pp* interaction vertices (PVs). At least one track should have $p_{\rm T}> 1.7~\text{GeV/}c$ and impact parameter (IP) *χ* ^2^ with respect to the primary interaction greater than 16. The IP *χ* ^2^ is defined as the difference between the *χ* ^2^ of the PV reconstructed with and without the considered track. A multivariate algorithm is used for the identification of secondary vertices consistent with the decay of a *b* hadron.

Simulated $B^{+} \to p \bar{p} K^{+}$ decays, generated uniformly in phase space, are used to optimize the signal selection and to evaluate the ratio of the efficiencies for each considered channel with respect to the *J*/*ψ* channel. Separate samples of $B^{+} \to J/\psi K^{+} \to p \bar{p} K^{+}$ and $B^{+} \to\eta_{c}(1S) K^{+} \to p \bar{p} K^{+}$ decays, generated with the known angular distributions, are used to check the dependence of the efficiency ratio on the angular distribution. In the simulation, *pp* collisions are generated using Pythia 6.4 [8] with a specific LHCb configuration [9]. Decays of hadronic particles are described by EvtGen [10] in which final state radiation is generated by Photos [11]. The interaction of the generated particles with the detector and its response are implemented using the Geant4 toolkit [12, 13] as described in Ref. [14].

## Candidate selection

Candidate $B^{+}\to p\bar{p} K^{+}$ decays are reconstructed from any combination of three charged tracks with total charge of +1. The final state particles are required to have a track fit with a $\chi ^{2}/{\rm ndf} < 3$ where ndf is the number of degrees of freedom. They must also have *p*>1500 MeV/*c*, $p_{\rm T} > 100~\text {MeV}/c$, and IP *χ* ^2^>1 with respect to any primary vertex in the event. Particle identification (PID) requirements, based on the RICH detector information, are applied to *p* and $\bar{p}$ candidates. The discriminating variables between different particle hypotheses (*π*, *K*, *p*) are the differences between log-likelihood values $\Delta\ln\mathcal{L}_{\alpha\beta}$ under particle hypotheses *α* and *β*, respectively. The *p* and $\bar{p}$ candidates are required to have $\Delta \ln\mathcal{L}_{p\pi}>-5$. The reconstructed *B* ^+^ candidates are required to have an invariant mass in the range 5079–5579 MeV/*c* ^2^. The asymmetric invariant mass range around the nominal *B* ^+^ mass is designed to select also $B^{+} \to p \bar{p} \pi^{+}$ candidates without any requirement on the PID of the kaon. The PV associated to each *B* ^+^ candidate is defined to be the one for which the *B* ^+^ candidate has the smallest IP *χ* ^2^. The *B* ^+^ candidate is required to have a vertex fit with a $\chi^{2}/{\rm ndf}<12$ and a distance greater than 3 mm, a *χ* ^2^ for the flight distance greater than 500, and an IP *χ* ^2^<10 with respect to the associated PV. The maximum distance of closest approach between daughter tracks has to be less than 0.2 mm. The angle between the reconstructed momentum of the *B* ^+^ candidate and the *B* ^+^ flight direction ($\theta_{\rm fl}$) is required to have $\cos\theta _{\rm fl}>0.99998$.

**Fig. 1:**
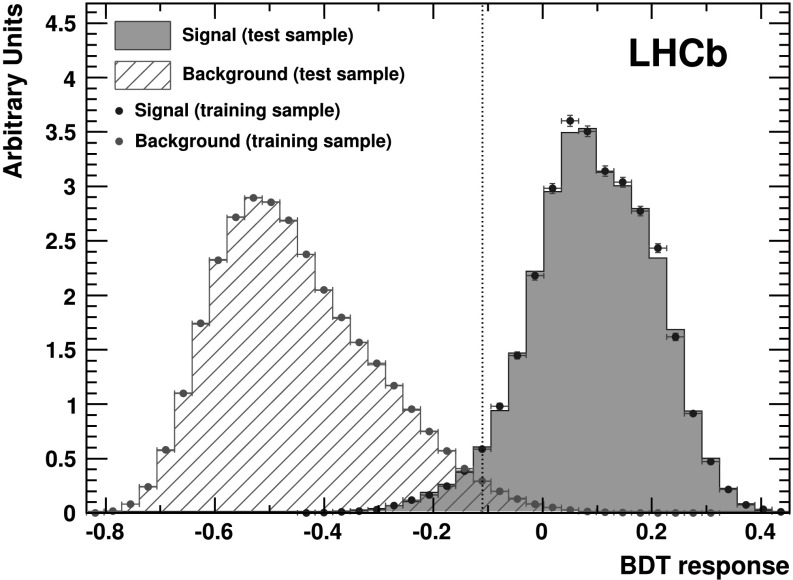
Distribution of the BDT algorithm response evaluated for background candidates from the data sidebands (*red hatched area*), and signal candidates from simulation (*blue filled area*). The *dotted line* (*black*) indicates the chosen BDT response value (Color figure online)

The reconstructed candidates that meet the above criteria are filtered using a boosted decision tree (BDT) algorithm [15]. The BDT is trained with a sample of simulated $B^{+} \to p \bar{p} K^{+}$ signal candidates and a background sample of data candidates taken from the invariant mass sidebands in the ranges 5080–5220 MeV/*c* ^2^ and 5340–5480 MeV/*c* ^2^. The variables used by the BDT to discriminate between signal and background candidates are: the $p_{\rm T}$ of each reconstructed track; the sum of the daughters’ *p*_*T*_; the sum of the IP *χ* ^2^ of the three daughter tracks with respect to the primary vertex; the IP of the daughter, with the highest $p_{\rm T}$, with respect to the primary vertex; the number of daughters with $p_{\rm T} > 900~\text {GeV}/c$; the maximum distance of closest approach between any two of the *B* ^+^ daughter particles; the IP of the *B* ^+^ candidate with respect to the primary vertex; the distance between primary and secondary vertices; the $\theta_{\rm fl}$ angle; the $\chi^{2}/{\rm ndf}$ of the secondary vertex; a pointing variable defined as $\frac{P\sin\theta}{P\sin\theta+ \sum_{i} p_{\rm T,i}}$, where *P* is the total momentum of the three-particle final state, *θ* is the angle between the direction of the sum of the daughter’s momentum and the direction of the flight distance of the *B* ^+^ and $\sum_{i} p_{{\rm T},i}$ is the sum of the transverse momenta of the daughters; and the log likelihood difference for each daughter between the assumed PID hypothesis and the pion hypothesis. The selection criterion on the BDT response (Fig. 1) is chosen in order to have a signal to background ratio of the order of unity. This corresponds to a BDT response value of −0.11. The efficiency of the BDT selection is greater than 92 % with a background rejection greater than 86 %.

## Signal yield determination

**Fig. 2:**
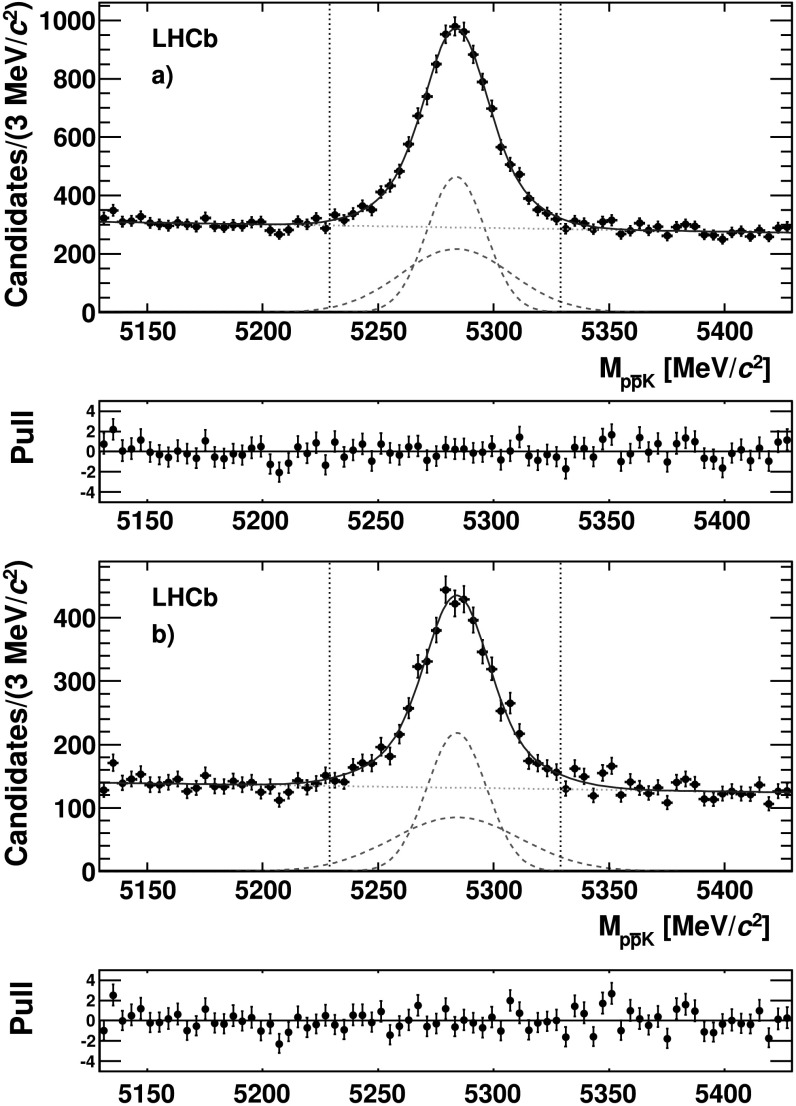
Invariant mass distribution of (**a**) all selected $B^{+} \to p \bar{p} K^{+}$ candidates and (**b**) candidates having $M_{p \bar {p}} < 2.85~\text {GeV}/c^{2}$. The *points with error* bars are the data and the *solid lines* are the result of the fit. The *dotted lines* represent the two Gaussian functions (*red*) and the dashed line the linear function (*green*) used to parametrize the signal and the background, respectively. The *vertical lines* (*black*) indicate the signal region. The two plots below the mass distributions show the pulls (Color figure online)

The signal yield is determined from an unbinned extended maximum likelihood fit to the invariant mass of selected $B^{+} \to p \bar{p} K^{+}$ candidates, shown in Fig. 2(a). The signal component is parametrized as the sum of two Gaussian functions with the same mean and different widths. The background component is parametrized as a linear function. The signal yield of the charmless component is determined by performing the same fit described above to the sample of $B^{+} \to p \bar{p} K^{+}$ candidates with $M_{p\bar{p}} < 2.85~\text {GeV}/c^{2}$, shown in Fig. 2(b). The *B* ^+^ mass and widths, evaluated with the invariant mass fits to all of the $B^{+} \to p \bar{p} K^{+}$ candidates, are compatible with the values obtained for the charmless component.

The signal yields for the charmonium contributions, $B^{+}\to(c\bar{c}) K^{+} \to p\bar{p} K^{+}$, are determined by fitting the $p\bar{p}$ invariant mass distribution of $B^{+} \to p\bar{p} K^{+}$ candidates within the *B* ^+^ mass signal window, $\vert M_{p\bar{p} K^{+}} - M_{B^{+}}\vert< 50~\text {MeV}/c^{2}$. Simulations show that no narrow structures are induced in the $p \bar{p}$ spectrum as kinematic reflections of possible $B^{+} \to p \bar{\varLambda} \to p \bar{p} K^{+}$ intermediate states.

**Fig. 3:**
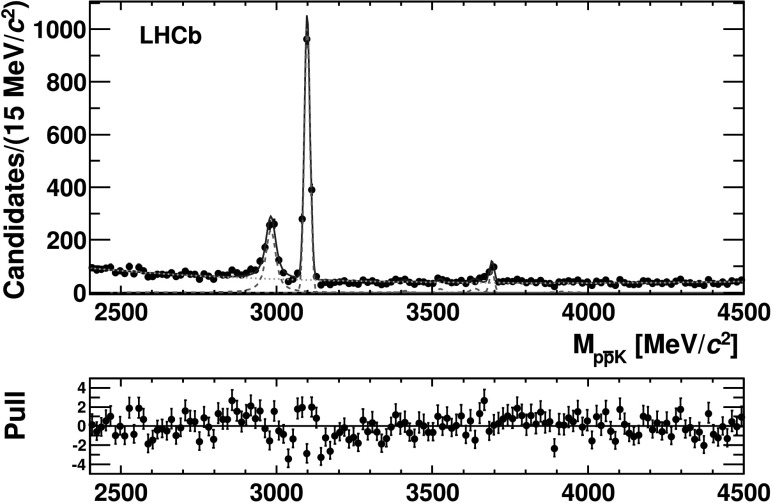
Invariant mass distribution of the $p \bar{p}$ system for $B^{+} \to p\bar{p} K^{+}$ candidates within the *B* ^+^ mass signal window, $\vert M(p\bar{p} K^{+}) - M_{B^{+}}\vert< 50~\text {MeV}/c^{2}$. The *dotted lines* represent the Gaussian and Voigtian functions (*red*) and the *dashed line* the smooth function (*green*) used to parametrize the signal and the background, respectively. The *bottom plot* shows the pulls (Color figure online)

An unbinned extended maximum likelihood fit to the $p\bar{p}$ invariant mass distribution, shown in Fig. 3, is performed over the mass range 2400–4500 MeV/*c* ^2^. The signal components of the narrow resonances *J*/*ψ*, *ψ*(2*S*), *h*_*c*_(1*P*), and *X*(3872), whose natural widths are much smaller than the $p\bar{p}$ invariant mass resolution, are parametrized by Gaussian functions. The signal components for the *η*_*c*_(1*S*), *χ*_*c*0_(1*P*), *η*_*c*_(2*S*), and *X*(3915) are parametrized by Voigtian functions.^2^ Since the $p\bar{p}$ invariant mass resolution is approximately constant in the explored range, the resolution parameters for all resonances, except the *ψ*(2*S*), are fixed to the *J*/*ψ* value (*σ*_*J*/*ψ*_=8.9±0.2 MeV/*c* ^2^). The background shape is parametrized as $f(M)=e^{c_{1}M+ c_{2}M^{2}}$ where *c*_1_ and *c*_2_ are fit parameters. The *J*/*ψ* and *ψ*(2*S*) resolution parameters, the mass values of the *η*_*c*_(1*S*), *J*/*ψ*, and *ψ*(2*S*) states, and the *η*_*c*_(1*S*) natural width are left free in the fit. The masses and widths for the other signal components are fixed to the corresponding world averages [16]. The $p\bar{p}$ invariant mass resolution, determined by the fit to the *ψ*(2*S*) is *σ*_*ψ*(2*S*)_=7.9±1.7 MeV/*c* ^2^.

**Fig. 4:**
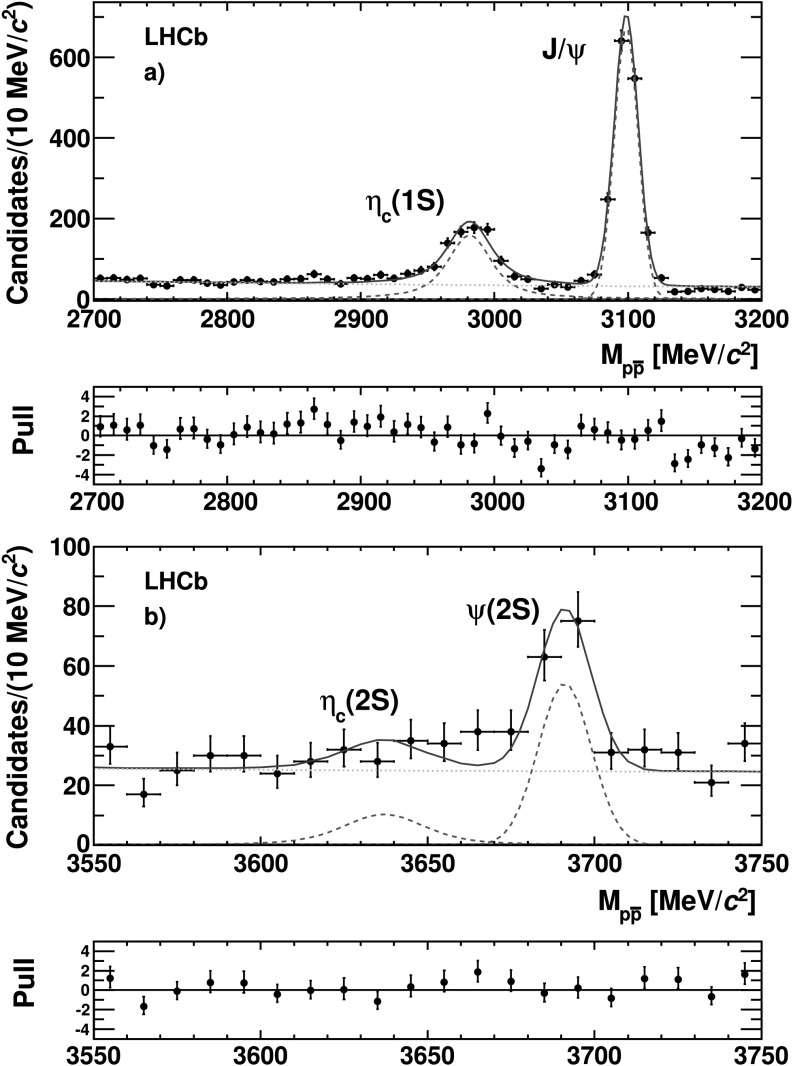
Invariant mass distribution of the $p \bar{p}$ system in the regions around (**a**) the *η*_*c*_(1*S*) and *J*/*ψ* and (**b**) the *η*_*c*_(2*S*) and *ψ*(2*S*) states. The *dotted lines* represent the Gaussian and the Voigtian functions (*red*) and the *dashed line* the smooth function (*green*) used to parametrize the signal and the background, respectively. The two plots below the mass distribution show the pulls (Color figure online)

**Fig. 5:**
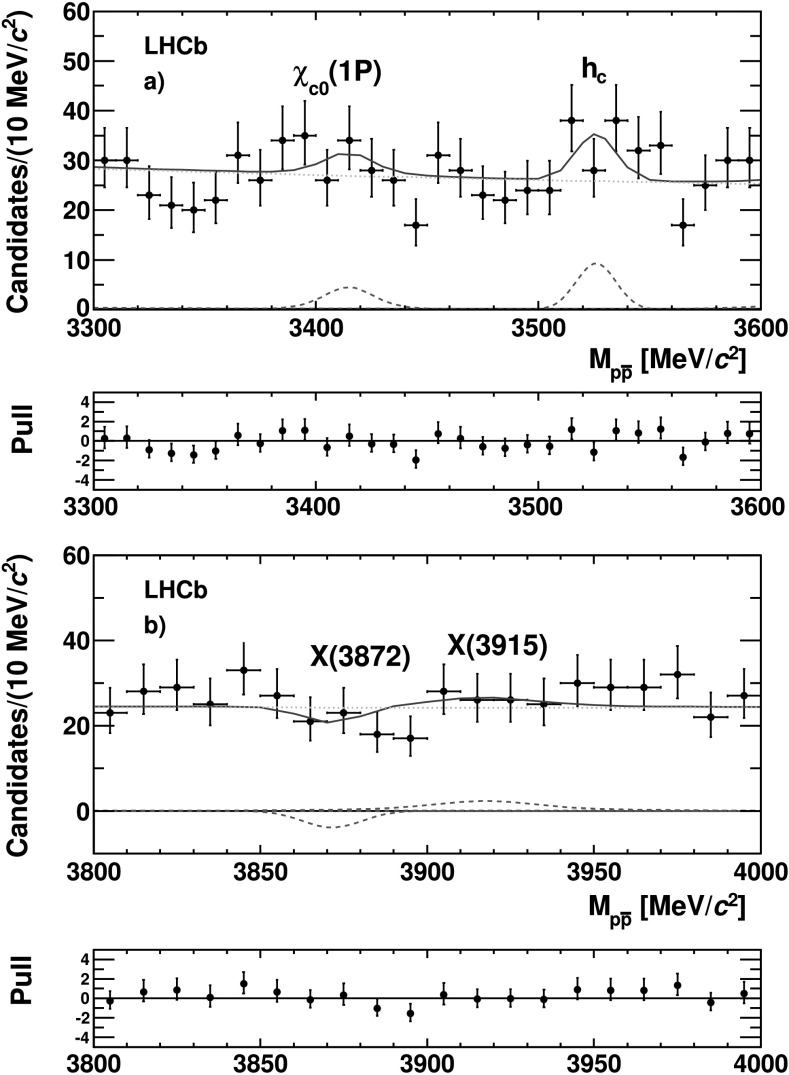
Invariant mass distribution of the $p \bar{p}$ system in the regions around (**a**) the *χ*_*c*0_(1*P*) and *h*_*c*_ and (**b**) the *X*(3872) and *X*(3915) states. The *dotted lines* represent the Gaussian and Voigitian functions (*red*) and the *dashed line* the smooth function (*green*) used to parametrize the signal and the background, respectively. The two plots below the mass distribution show the pulls (Color figure online)

The fit result is shown in Fig. 3. Figures 4 and 5 show the details of the fit result in the regions around the *η*_*c*_(1*S*) and *J*/*ψ*, *η*_*c*_(2*S*) and *ψ*(2*S*), *χ*_*c*0_(1*P*) and *h*_*c*_(1*P*), and *X*(3872) and *X*(3915) resonances. Any bias introduced by the inaccurate description of the tails of the *η*_*c*_(1*S*), *J*/*ψ* and *ψ*(2*S*) resonances is taken into account in the systematic uncertainty evaluation.

The contribution of $c\bar{c}\to p\bar{p}$ from processes other than $B^{+} \to p \bar{p} K^{+}$ decays, denoted as “non-signal”, is estimated from a fit to the $p \bar{p}$ mass in the *B* ^+^ mass sidebands 5130–5180 and 5380–5430 MeV/*c* ^2^. Except for the *J*/*ψ* mode, no evidence of a non-signal contribution is found. The non-signal contribution to the *J*/*ψ* signal yield in the *B* ^+^ mass window is 43±11 candidates and is subtracted from the number of *J*/*ψ* signal candidates.

The signal yields, corrected for the non-signal contribution, are reported in Table 1. For the intermediate charmonium states *η*_*c*_(2*S*), *χ*_*c*0_(1*P*), *h*_*c*_(1*P*), *X*(3872) and *X*(3915), there is no evidence of signal. The 95 % CL upper limits on the number of candidates are shown in Table 1 and are determined from the likelihood profile integrating over the nuisance parameters. Since for the *X*(3872) the fitted signal yield is negative, the upper limit has been calculated integrating the likelihood only in the physical region of a signal yield greater than zero.

**Table 1:** Signal yields for the different channels and corresponding 95 % CL upper limits for modes with less than 3*σ* statistical significance. For the *J*/*ψ* mode, the non-signal yield is subtracted. Uncertainties are statistical only

*B* ^+^ decay mode	Signal yield	Upper limit (95 % CL)
$p\bar{p} K^{+}$ [total]	6951 ± 176	
$p\bar{p} K^{+}\ [M_{p \bar {p}} < 2.85~\text {GeV}/c^{2}]$	3238 ± 122	
*J*/*ψK* ^+^	1458 ± 42	
*η*_*c*_(1*S*)*K* ^+^	856 ± 46	
*ψ*(2*S*)*K* ^+^	107 ± 16	
*η*_*c*_(2*S*)*K* ^+^	39 ± 15	<65.4
*χ*_*c*0_(1*P*)*K* ^+^	15 ± 13	<38.1
*h*_*c*_(1*P*)*K* ^+^	21 ± 11	<40.2
*X*(3872)*K* ^+^	−9 ± 8	<10.3
*X*(3915)*K* ^+^	13 ± 17	<42.1

## Efficiency determination

The ratio of branching fractions is calculated using

2 $$\begin{aligned} \mathcal{R}({\rm mode}) =&\frac{\mathcal{B}(B^{+} \to{\rm mode}\to p\bar{p} K^{+})}{\mathcal{B}(B^{+}\to J/\psi K^{+}\to p\bar{p} K^{+})} \\=& \frac {N_{\rm mode}}{N_{ J/\psi }}\times \frac{\epsilon_{ J/\psi }}{\epsilon_{\rm mode}}, \end{aligned}$$

where $N_{\rm mode}$ and *N*_*J*/*ψ*_ are the signal yields for the given mode and the reference mode, $B^{+}\to J/\psi K^{+}\to p\bar{p} K^{+}$, and $\epsilon_{\rm mode}/\epsilon_{ J/\psi }$ is the corresponding ratio of efficiencies. The efficiency is the product of the reconstruction, trigger, and selection efficiencies, and is estimated using simulated data samples.

**Fig. 6:**
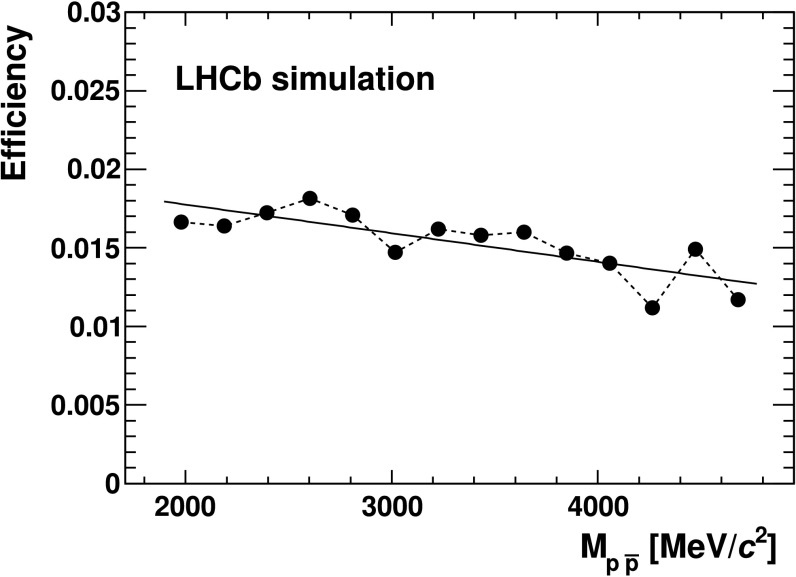
Efficiency as a function of $M_{p \bar{p}}$ for $B^{+} \to p \bar{p} K^{+}$ decays. The *solid line* represents the linear fit to the efficiency distribution; the *dashed line* is the point-by-point interpolation used to estimate the systematic uncertainty

Since the track multiplicity distribution for simulated events differs from that observed in data, simulated candidates are assigned a weight so that the weighted distribution reproduces the observed multiplicity distribution. The distributions of $\Delta\ln\mathcal{L}_{K\pi}$ and $\Delta\ln\mathcal{L}_{p\pi}$ for kaons and protons in data are obtained in bins of momentum, pseudorapidity and number of tracks from control samples of *D* ^∗+^→*D* ^0^(→*K* ^−^ *π* ^+^)*π* ^+^ decays for kaons and *Λ*→*pπ* ^−^ decays for protons, which are then used on a track-by-track basis to correct the simulation. The efficiency as a function of $M_{p \bar{p}}$ is shown in Fig. 6. A linear fit to the efficiency distribution is performed and the efficiency ratios are determined based on the fit result.

## Systematic uncertainties

The measurements of the relative branching fractions depend on the ratios of signal yields and efficiencies with respect to the reference mode. Since the final state is the same in all cases, most of the systematic uncertainties cancel. The systematic uncertainty on the efficiency ratio, in each region of $p \bar{p}$ invariant mass, is determined from the difference between the efficiency ratios calculated using the solid fitted line and the dashed point-by-point interpolation shown in Fig. 6. The uncertainty associated with the evaluation of the *B* ^+^ signal yield has been determined by varying the fit range by ±30 MeV/*c* ^2^, using a single Gaussian instead of a double Gaussian function to model the signal PDF, and using an exponential function to model the background. For each charmonium resonance the systematic uncertainty on the signal yield has been investigated by varying the *B* mass signal window by ±10 MeV/*c* ^2^, the signal and background shape parametrization and the subtraction of the $c\bar{c}$ contribution from the continuum. The systematic uncertainty associated with the parametrization of the signal tails of the *J*/*ψ*, *η*_*c*_(1*S*) and *ψ*(2*S*) resonances is taken into account by taking the difference between the number of candidates in the observed distribution and the number of candidates calculated from the integral of the fit function in the range −6*σ* to −2.5*σ*. The systematic uncertainty associated with the selection procedure is estimated by changing the value of the BDT selection to −0.03, which retains 85 % of the signal with a 30 % background, and is found to be negligible. The contributions to the systematic uncertainties from the different sources are listed in Table 2. The total systematic uncertainty is determined by adding the individual contributions in quadrature.

**Table 2:** Relative systematic uncertainties (in %) on the relative branching fractions from different sources. The total systematic uncertainty is determined by adding the individual contributions in quadrature

Source	$\mathcal{R}({\rm total})$	$\mathcal{R}(M_{p \bar {p}} < 2.85~\text {GeV}/c^{2}) $	$\mathcal{R}( \eta _{ c } (1S))$	$\mathcal{R}(\psi(2S))$
Efficiency ratio	0.21	0.5	3.3	4.8
*B* ^+^ mass fit range	0.16	0.5	–	–
Sig. and Bkg. shape	2.5	3.6	1.8	6.5
*B* ^+^ mass window	0.6	0.6	0.9	3.8
Non-signal component	–	–	0.4	5.1
Signal tail param.	1.0	1.0	1.2	4.3
Total	2.8	3.8	4.1	11.3

Source	$\mathcal{R}(\eta_{c}(2S))$	$\mathcal{R}(\chi_{c0}(1P))$	$\mathcal{R}(h_{c}(1P))$	$\mathcal{R}(X(3872))$	$\mathcal {R}(X(3915))$
Efficiency ratio	4.4	2.5	3.4	6.5	7.0
*B* ^+^ mass fit range	–	–	–	–	
Sig. and Bkg. shape	3.9	3.3	14.3	5.6	10.1
*B* ^+^ mass window	11.3	23.6	23.6	17.5	7.5
Non-signal component	–	–	–	–	–
Signal tail param.	1.0	1.0	1.0	1.0	1.0
Total	12.8	24.0	27.8	19.5	15.5

## Results

The results are summarized in Table 3 and the values of the product of branching fractions derived from our measurement using the world average values $\mathcal{B}(B^{+} \to J/\psi K^{+}) =(1.013\pm0.034)\times10^{-3}$ and $\mathcal{B}(J/\psi\to p\bar{p}) =(2.17\pm0.07)\times10^{-3}$ [16] are listed in Table 4. The branching fractions obtained are compatible with the world average values [16]. The upper limit on $\mathcal{B}(B^{+} \to\chi_{c0}(1P) K^{+} \to p \bar{p} K^{+})$ is compatible with the world average $\mathcal{B}(B^{+} \to\chi _{c0}(1P) K^{+}) \times\mathcal{B}(\chi_{c0}(1P) \to p \bar{p}) = (0.030 \pm0.004) \times 10^{-6}$ [16]. We combine our upper limit for *X*(3872) with the known value for $\mathcal{B} (B^{+} \to X(3872) K^{+} ) \times\mathcal{B} (X(3872) \to J/\psi\pi^{+} \pi^{-})= (8.6 \pm0.8) \times10^{-6}$ [16] to obtain the limit

$$ \frac{\mathcal{B} (X(3872) \to p \bar{p})}{\mathcal{B} (X(3872) \to J/\psi\pi^{+} \pi^{-})}< 2.0\times10^{-3}. $$

This limit challenges some of the predictions for the molecular interpretations of the *X*(3872) state and is approaching the range of predictions for a conventional *χ*_*c*1_(2*P*) state [17, 18]. Using our result and the *η*_*c*_(2*S*) branching fraction $\mathcal{B} (B^{+} \to\eta_{c}(2S) K^{+})\times \mathcal{B} (\eta_{c}(2S) \to K \bar{K} \pi) = (3.4\, ^{+2.3}_{-1.6}) \times10^{-6}$ [16], a limit of

$$ \frac{\mathcal{B} (\eta_{c}(2S) \to p \bar{p})}{\mathcal{B} (\eta_{c}(2S) \to K \bar{K} \pi)} < 3.1 \times10^{-2} $$

is obtained.

**Table 3:** Signal yields, efficiency ratios, ratios of branching fractions and corresponding upper limits

$B^{+}\to({\rm mode})$ $\to p\bar{p} K^{+}$	Yield ± stat ± syst	$\epsilon_{\rm mode}/\epsilon_{ J/\psi }$ ± syst	$\mathcal{R}({\rm mode})$ ± stat ± syst	Upper Limit 95 % CL
*J*/*ψK* ^+^	1458±42±24	–	1	–
total	6951±176±171	0.970±0.002	4.91±0.19±0.14	–
${M_{p \bar {p}} < 2.85~\text {GeV}/c^{2}}$	3238±122±121	1.097±0.006	2.02±0.10±0.08	–
*η*_*c*_(1*S*)*K* ^+^	856±46±19	1.016±0.034	0.578±0.035±0.026	–
*ψ*(2*S*)*K* ^+^	107±16±13	0.921±0.044	0.080±0.012±0.009	–
*η*_*c*_(2*S*)*K* ^+^	39±15±5	0.927±0.041	0.029±0.011±0.004	<0.048
*χ*_*c*0_(1*P*)*K* ^+^	15±13±4	0.957±0.024	0.011±0.009±0.003	<0.028
*h*_*c*_(1*P*)*K* ^+^	21±11±5	0.943±0.032	0.015±0.008±0.004	<0.029
*X*(3872)*K* ^+^	−9±8±2	0.896±0.058	−0.007±0.006±0.002	<0.008
*X*(3915)*K* ^+^	13±17±5	0.890±0.062	0.010±0.013±0.002	<0.032

**Table 4:** Branching fractions for $B^{+}\to({\rm mode})\to p\bar{p} K^{+}$ derived using the world average value of the $\mathcal{B}(B^{+}\to J/\psi K^{+})$ and $\mathcal{B}( J/\psi \to p\bar{p})$ branching fractions [16]. For the charmonium modes we compare our values to the product of the independently measured branching fractions. The first uncertainties are statistical, the second systematic in the present measurement, and the third systematic from the uncertainty on the *J*/*ψ* branching fraction

*B* ^+^ decay mode	$\mathcal{B}(B^{+}\to({\rm mode})\to p\bar{p} K^{+})$ (×10^6^)	UL (95 % CL) (×10^6^)	Previous measurements (×10^6^) [4, 5]
total	10.81±0.42±0.30±0.49		$10.76^{+0.36}_{-0.33} \pm0.70$
${M_{p \bar {p}} < 2.85~\text {GeV}/c^{2}}$	4.46±0.21±0.18±0.20		5.12±0.31
*η*_*c*_(1*S*)*K* ^+^	1.27±0.08±0.05±0.06		1.54±0.16
*ψ*(2*S*)*K* ^+^	0.175±0.027±0.020±0.008		0.176±0.012
*η*_*c*_(2*S*)*K* ^+^	0.063±0.025±0.009±0.003	<0.106	
*χ*_*c*0_(1*P*)*K* ^+^	0.024±0.021±0.006±0.001	<0.062	0.030±0.004
*h*_*c*_(1*P*)*K* ^+^	0.034±0.018±0.008±0.002	<0.064	
*X*(3872)*K* ^+^	−0.015±0.013±0.003±0.001	<0.017	
*X*(3915)*K* ^+^	0.022±0.029±0.004±0.001	<0.071	

## Summary

Based on a sample of 6951±176 $B^{+} \to p \bar{p} K^{+}$ decays reconstructed in a data sample, corresponding to an integrated luminosity of 1.0 fb^−1^, collected with the LHCb detector, the following relative branching fractions are measured  An upper limit on the ratio $\frac{\mathcal{B} (B^{+} \to X(3872) K^{+} \to p \bar{p} K^{+})}{\mathcal{B}(B^{+} \to J/\psi K^{+} \to p \bar{p} K^{+})} < 0.017$ is obtained, from which a limit of

$$ \frac{\mathcal{B} (X(3872) \to p \bar{p})}{\mathcal{B} (X(3872) \to J/\psi\pi^{+} \pi^{-})}< 2.0\times10^{-3} $$

is derived.

## Footnotes

1 The inclusion of charge-conjugate modes is implied throughout the paper.

2 A Voigtian function is the convolution of a Breit-Wigner function with a Gaussian distribution.
